# A Quasi-Monte Carlo Method Based on Neural Autoregressive Flow

**DOI:** 10.3390/e27090952

**Published:** 2025-09-13

**Authors:** Yunfan Wei, Wei Xi

**Affiliations:** 1 School of Mathematics, South China University of Technology, Guangzhou 510641, China; 2 School of Statistics, Beijing Normal University, Beijing 100875, China

**Keywords:** transport map, quasi-Monte Carlo, normalizing flow, autoregressive model, stock return prediction

## Abstract

This paper proposes a novel transport quasi-Monte Carlo framework that combines randomized quasi-Monte Carlo sampling with a neural autoregressive flow architecture for efficient sampling and integration over complex, high-dimensional distributions. The method constructs a sequence of invertible transport maps to approximate the target density by decomposing it into a series of lower-dimensional marginals. Each sub-model leverages normalizing flows parameterized via monotonic beta-averaging transformations and is optimized using forward Kullback–Leibler (KL) divergence. To enhance computational efficiency, a hidden-variable mechanism that transfers optimized parameters between sub-models is adopted. Numerical experiments on a banana-shaped distribution demonstrate that this new approach outperforms standard Monte Carlo-based normalizing flows in both sampling accuracy and integral estimation. Further, the model is applied to A-share stock return data and shows reliable predictive performance in semiannual return forecasts, while accurately capturing covariance structures across assets. The results highlight the potential of transport quasi-Monte Carlo (TQMC) in financial modeling and other high-dimensional inference tasks.

## 1. Introduction

### 1.1. Quasi-Monte Carlo

For a wide range of problems in quantitative finance such as option pricing under stochastic volatility, portfolio risk estimation, Value-at-Risk (VaR) and Expected Shortfall (ES) computation, and optimal asset allocation under uncertainty, one often needs to compute expectations with respect to high-dimensional probability distributions. These expectations can typically be rewritten as integrals over a d-dimensional Gaussian or log-normal measure, where d reflects the number of underlying assets, discretized time steps, and model complexity. In realistic settings, d can reach into the hundreds or even thousands. Under such conditions, classical numerical quadrature methods, such as Gauss–Hermite or sparse grid integration, suffer from the curse of dimensionality and become computationally intractable [1].

To overcome this barrier, the Monte Carlo (MC) method has become a cornerstone of computational finance. Originally developed by Ulam and von Neumann during the Manhattan Project and later formalized for integration by Metropolis and Rosenbluth, MC methods use random sampling to approximate integrals and expectations. Their convergence rate, $O(N^{-1/2})$, is independent of dimension, making MC particularly attractive in high-dimensional settings [2]. MC methods have been applied to simulate geometric Brownian motion and price path-dependent derivatives such as Asian and barrier options, and to compute Greeks via pathwise or likelihood ratio methods [3,4]. The method’s generality and flexibility allow it to accommodate non-smooth payoffs, stochastic volatility, jump diffusion models, and more.

However, a major drawback of the MC method is its slow convergence: reducing the standard error by a factor of 10 requires 100 times more samples. This computational inefficiency becomes especially pronounced when simulating rare events or computing tail risk measures. To address this limitation, the field has increasingly turned to quasi-Monte Carlo (QMC) methods. QMC replaces random sampling with low-discrepancy sequences: deterministic point sets that fill the integration domain more uniformly than purely random points [5,6]. Common sequences include Sobol’, Halton, and Faure sequences. For sufficiently smooth integrands, QMC achieves a convergence rate of $O(N^{-1}{\left(\log N\right)}^{d})$, which is asymptotically faster than MC for fixed dimension. Applications in finance have demonstrated the superior efficiency of QMC in pricing exotic options, computing risk measures, and solving portfolio optimization problems [7,8].

Nonetheless, QMC’s error is deterministic and thus lacks a statistical interpretation. Moreover, its performance is sensitive to the dimension and smoothness of the integrand, and error bounds depend on complex measures like variation in the sense of Hardy and Krause. To remedy this, Randomized Quasi-Monte Carlo (RQMC) methods were developed which combine the structure of QMC with randomization (e.g., digital scrambling, shifting, or lattice shifting) to generate unbiased estimators [9]. This allows for standard error estimation and the use of variance-based stopping rules while retaining the variance reduction benefits of QMC. RQMC has been particularly successful in financial simulations with smooth payoff structures and moderate effective dimension, as demonstrated in the pricing of mortgage-backed securities, insurance products, and risk aggregation models [3,10,11].

Despite their success, MC, QMC, and RQMC methods often rely on the assumption that the target distribution is known in closed form, at least up to a normalizing constant. This enables efficient sampling and importance weighting. However, in modern finance and econometrics, models are increasingly complex, with intractable or implicit densities as in latent variable models, generative stochastic networks, or when modeling real-world asset returns under heavy tails and asymmetries. In such contexts, classical sampling and quadrature approaches fail to scale or adapt. To address this, this paper proposes a learning-based method that estimates the target distribution through a transport map: a deterministic, invertible transformation that pushes forward a simple base distribution (such as a uniform distribution on the unit hypercube) onto the target distribution.

### 1.2. Normalizing Flows and Neural Autoregressive Flow

Normalizing flows (NFs) are a class of powerful generative models designed to model complex probability distributions through a sequence of invertible, differentiable transformations developed through the progress of machine learning techniques. Given a base distribution, typically a standard multivariate Gaussian or uniform distribution, NFs construct a bijective mapping to a target distribution via the change-of-variables formula, which allows for exact and tractable computation of the density function and efficient sampling. The elegant mathematical structure gives NFs several desirable properties over other generative models, including tractable likelihood evaluation and efficient sample generation.

The foundational theory behind NFs emerged from the field of optimal transport and statistical mechanics, particularly in the work of Tabak and Vanden-Eijnden (2010) [12], who introduced a method for transforming probability densities using transport maps governed by differential equations. Later, Tabak and Turner (2013) [13] generalized this approach into a family of density estimators based on nonparametric optimal transport, proposing an iterative learning mechanism for constructing transport maps via convex optimization and regularization. Normalizing flows offer a powerful framework for density modeling and variational inference, using compositions of invertible transformations to model complex distributions while enabling exact likelihood computation and efficient sampling [14,15]. These works laid the theoretical foundation for using invertible maps to perform density estimation and generation.

Early applications of NFs demonstrated their potential across tasks such as clustering, classification, and nonlinear dimensionality reduction. For example, Agnelli et al. (2010) [16] explored using transformations for feature-space clustering. Subsequently, Rippel and Adams (2013) [17] investigated NFs for high-dimensional density estimation, showing that NFs could transform structured, real-world datasets into simple latent representations that are more amenable to analysis and modeling.

A major breakthrough came with the introduction of NFs to variational inference by Rezende and Mohamed (2015) [15]. In their work, they introduced Planar and Radial Flows, simple invertible transformations that enhanced the expressiveness of variational posteriors in Variational Autoencoders (VAEs). These flows enabled gradient-based optimization and reparameterization while preserving tractable density evaluation, addressing a key limitation in standard VAEs, where the posterior is often overly simplistic (e.g., mean-field Gaussian).

In parallel, Dinh et al. (2014, 2016) introduced the NICE and RealNVP models [18], which popularized the use of coupling layers—a type of invertible transformation with triangular Jacobians enabling efficient computation of both forward transformations and Jacobian determinants. These architectures supported the high-capacity modeling of complex data such as images and audio, and became widely adopted due to their simplicity and tractability.

Building on these developments, the field progressed to more expressive architectures such as autoregressive flows. These models decompose a joint distribution into a sequence of conditionals, enabling expressive modeling of dependencies across dimensions. A notable instance is the Inverse Autoregressive Flow (IAF) by Kingma et al. (2016) [19], which conditions transformations on an auxiliary autoregressive network such as MADE (Masked Autoencoder for Distribution Estimation; [20]). IAFs preserve fast parallel sampling from the approximate posterior, making them suitable for training large-scale VAEs. However, IAFs rely on affine transformations, which may be insufficient to represent distributions with highly nonlinear structure unless many layers are stacked, resulting in deep and computationally expensive architectures.

To address the limitations of affine transformations in autoregressive flows, Huang et al. (2018) introduced the neural autoregressive flow (NAF) [21]. In NAF, each affine transformation is replaced with a strictly monotonic neural network, allowing for a universal approximation of continuous distributions under mild assumptions. The model preserves the autoregressive structure of IAF while greatly enhancing flexibility. However, NAF introduces an architectural bottleneck: each transformation is governed by a separate transformer network whose parameters are predicted by a distinct conditioner network, resulting in a quadratic parameter complexity in terms of model width. While this structure improves expressiveness, it can hinder scalability, particularly in high-dimensional settings where memory and computation are constrained.

Various techniques have been proposed to reduce the redundancy and inefficiency of this dual-network architecture. For instance, conditional weight normalization and sparsity regularization [22] help mitigate overparameterization. However, these fixes only partially resolve the scalability issues inherent in NAF’s design. In response, De Cao, Aziz, and Titov (2019) proposed Block Neural Autoregressive Flow (B-NAF) [23], which unifies the conditioner and transformer into a single feedforward neural network with carefully structured block-wise transformations. B-NAF achieves similar expressiveness to NAF while drastically reducing parameter count, training time, and memory usage. This innovation allows for tractable high-dimensional density estimation with improved computational efficiency, making B-NAF a practical candidate for tasks such as likelihood-based generative modeling, Bayesian posterior estimation, and density-based clustering in complex domains.

Overall, the development of normalizing flows represents a confluence of deep learning, probability theory, and numerical analysis, providing a flexible and theoretically grounded framework for density modeling and transformation-based sampling. Their applications now span generative modeling, Bayesian inference, density ratio estimation, and scientific computing, demonstrating wide applicability beyond the original scope of variational inference.

Inspired by the development of NAF and the transport map, a quasi-Monte Carlo method based on an NAF architecture is proposed. This idea builds on optimal transport theory and normalizing flows, particularly the neural autoregressive flow (NAF) model introduced by Huang et al. [21]. By learning such a map from data or samples, this paper aims to generalize QMC-type integration to implicitly defined distributions, combining function approximation with deterministic sampling to handle high-dimensional, non-Gaussian, and irregular financial distributions.

Building on the developments in normalizing flows and inspired in particular by the NAF architecture, the next section introduces a novel quasi-Monte Carlo (QMC) method guided by transport maps parameterized through NAF-like structures. This approach leverages the core insight from optimal transport theory: that complex distributions can be obtained by deterministically pushing forward a simple base distribution—such as the uniform distribution on the unit hypercube—through a sequence of invertible transformations across dimensions. By training a transport map using neural autoregressive architectures, this method effectively learns to approximate the target distribution from data or samples, even when its density is analytically intractable. The resulting map allows us to integrate quasi-Monte Carlo techniques within this learned representation. This fusion of function approximation and low-discrepancy sampling extends QMC methods to settings with implicitly defined, non-Gaussian, and high-dimensional target distributions—common in modern financial modeling—thus bridging the gap between classical numerical integration and deep generative modeling.

## 2. Methodology

### 2.1. Neural Autoregressive Flow Architecture

**Figure 1:**
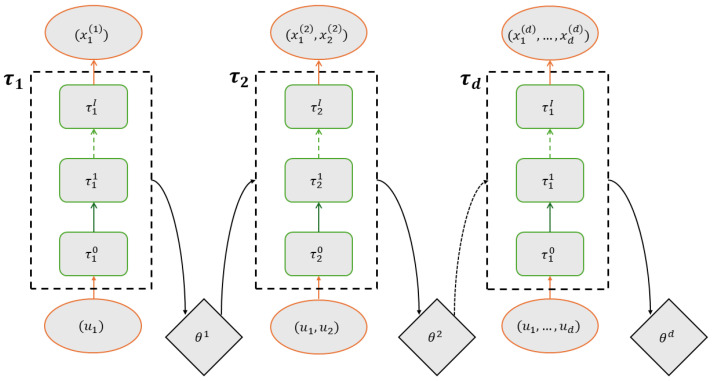
Neural autoregressive flow architecture.

Suppose $(u_{1},\ldots ,u_{d})∼$$Unif({\left[0,1\right]}^{d})$ is the *d* dimensional generated from RQMC. Our goal is to design an autoregressive flow to map $\left(u_{1},\ldots ,u_{d}\right)\to \left(x_{1},\ldots ,x_{d}\right)$, such that $(x_{1},\ldots ,x_{d})∼P$. *P* is a target distribution defined on $R^{d}$ with density function $p(x_{1},\ldots ,x_{d})$. To achieve this, a neural autoregressive flow (NAF) based on the architecture in [21] is built. The overview is shown in Figure 1. This architecture consists of *d* individual neural network sub-models. The *k*-th sub-model takes the input of the first *k*-dimension samples $\left(u_{1},\ldots ,u_{k}\right)$ and a hidden variable from the k−1-th model and aims to approximate the marginal distribution of the target distribution *P* on its first *k* dimensions, denoted by $p_{k}$. The hidden variable will be explained later. The *k*-th sub-model pushes the inputs $\left(u_{1},\ldots ,u_{k}\right)$ through I+1 reversible transport maps $\tau_{k}^{0},\tau_{k}^{1},\ldots ,\tau_{k}^{I}$ to obtain a set of samples $\left(x_{1}^{\left(k\right)},\ldots ,x_{k}^{\left(k\right)}\right)=\tau_{k}\left(u_{1},\ldots ,u_{k}\right)$, where $\tau_{k}=\tau_{k}^{I}\circ \tau_{k}^{I-1}\circ \ldots \circ \tau_{k}^{1}\circ \tau_{k}^{0}$ is the composition of all I+1 mappings. $\tau_{k}$ is as defined in normalizing flows in the literature [13]. The density of $\left(x_{1}^{\left(k\right)},\ldots ,x_{k}^{\left(k\right)}\right)$, which is induced by the transport maps, can be written as

$$\begin{array}{cc} q_{\tau_{k}}\left(x_{1}^{\left(k\right)},\ldots ,x_{k}^{\left(k\right)}\right) & ={|detJ_{\tau_{k}}\left(u_{1},\ldots ,u_{k}\right)|}^{-1}\\ \; & ={|detJ_{\tau_{k}}\left({\tau_{k}}^{-1}\left(x_{1}^{\left(k\right)},\ldots ,x_{k}^{\left(k\right)}\right)\right)|}^{-1}, \end{array}$$

where $J_{\tau_{k}}\left(u_{1},\ldots ,u_{k}\right)={\left(\frac{\partial \tau_{k}\left(u_{i}\right)}{\partial u_{j}}\right)}_{\left(k\times k\right)},1\leq i,j\leq k$ is the k×k Jacobian matrix of the projection $\tau_{k}$.

To stimulate the training, the model uses forward Kullback–Leibler (KL) divergence as the objective function to measure the difference between the target and approximated distribution in the *k*-th sub-model:

$$\begin{array}{cc} \mathrm{KL}(q_{\tau_{k}}∥p_{k})\; & =\int q_{\tau_{k}}\left(\tau_{k}\left(u_{1},\ldots ,u_{k}\right)\right)\log\frac{q_{\tau_{k}}\left(\tau_{k}\left(u_{1},\ldots ,u_{k}\right)\right)}{p_{k}\left(\tau_{k}\left(u_{1},\ldots ,u_{k}\right)\right)}\;du_{1},\ldots ,du_{k},\\ \; & =\int \log\frac{q_{\tau_{k}}\left(x_{1}^{\left(k\right)},\ldots x_{k}^{\left(k\right)}\right)}{p_{k}\left(x_{1}^{\left(k\right)},\ldots ,x_{k}^{\left(k\right)}\right)}\;dx_{1}^{\left(k\right)},\ldots ,dx_{k}^{\left(k\right)}\\ \; & =\frac{1}{N}\sum_{n=1}^{N}\log\frac{q_{\tau_{k}}\left(x_{n1}^{\left(k\right)},\ldots ,x_{nk}^{\left(k\right)}\right)}{p_{k}\left(x_{n1}^{\left(k\right)},\ldots ,x_{nk}^{\left(k\right)}\right)} \end{array}$$

$$\begin{array}{cc} \mathrm{KL}(q_{\tau_{k}}∥p_{k})\; & =\int q_{\tau_{k}}\left(\tau_{k}\left(u_{1},\ldots ,u_{k}\right)\right)\log\frac{q_{\tau_{k}}\left(\tau_{k}\left(u_{1},\ldots ,u_{k}\right)\right)}{p_{k}\left(\tau_{k}\left(u_{1},\ldots ,u_{k}\right)\right)}\;du_{1},\ldots ,du_{k},\\ \; & =\int \log\frac{q_{\tau_{k}}\left(x_{1}^{\left(k\right)},\ldots ,x_{k}^{\left(k\right)}\right)}{p_{k}\left(x_{1}^{\left(k\right)},\ldots ,x_{k}^{\left(k\right)}\right)}\;dx_{1}^{\left(k\right)},\ldots ,dx_{k}^{\left(k\right)} \end{array}$$

A numerical calculation of the KL divergence from the trained samples would be $\frac{1}{N}\sum_{n=1}^{N}\log\frac{q_{\tau_{k}}\left(x_{n1}^{\left(k\right)},\ldots ,x_{nk}^{\left(k\right)}\right)}{p_{k}\left(x_{n1}^{\left(k\right)},\ldots ,x_{nk}^{\left(k\right)}\right)}$. *N* is the total number of training data, and $\left(x_{n1}^{\left(k\right)},\ldots ,x_{nk}^{\left(k\right)}\right)=\tau_{k}\left(u_{n1},\ldots ,u_{nk}\right)$ is the *n*-th trained sample from the *k*-th sub-model. Using the composition $\tau_{k}=\tau_{k}^{I}\circ \tau_{k}^{I-1}\circ \ldots \circ \tau_{k}^{1}\circ \tau_{k}^{0}$, there is a sequence of samples during the transport maps:

$$\begin{array}{cc} y_{0}^{\left(k\right)} & =\tau_{k}^{0}\left(u_{1},\ldots ,u_{k}\right)\\ y_{1}^{\left(k\right)} & =\tau_{k}^{1}\left(y_{0}^{\left(k\right)}\right)\\ \; & \vdots \\ \left(x_{1}^{\left(k\right)},\ldots ,x_{k}^{\left(k\right)}\right)=y_{I}^{\left(k\right)} & =\tau_{k}^{I}\left(y_{I-1}^{\left(k\right)}\right) \end{array}$$

$y_{i}^{\left(k\right)}$ is a *k*-dimension variable generated after the transport map $\tau_{k}^{i}$. With this sequence of midway samples and applying the chain rule, the objective function becomes

$$\begin{array}{cc} \mathrm{KL}(q_{\tau_{k}}∥p_{k})\; & =\frac{1}{N}\sum_{n=1}^{N}\log\frac{q_{\tau_{k}}\left(x_{n1}^{\left(k\right)},\ldots ,x_{nk}^{\left(k\right)}\right)}{p_{k}\left(x_{n1}^{\left(k\right)},\ldots ,x_{nk}^{\left(k\right)}\right)}\\ \; & =\frac{1}{N}\sum_{n=1}^{N}\left[\log q_{\tau_{k}}\left(x_{n1}^{\left(k\right)},\ldots ,x_{nk}^{\left(k\right)}\right)-\log p_{k}\left(x_{n1}^{\left(k\right)},\ldots ,x_{nk}^{\left(k\right)}\right)\right]\\ \; & =\frac{1}{N}\sum_{n=1}^{N}[-\log\prod_{i=0}^{I}|detJ_{\tau_{k}^{i}}\left(y_{n,(i-1)}^{\left(k\right)}\right)\left|-\log p_{k}\left(x_{n1}^{\left(k\right)},\ldots ,x_{nk}^{\left(k\right)}\right)\right] \end{array}$$

$J_{\tau_{k}^{i}}\left(y_{n,(i-1)}^{\left(k\right)}\right)=\frac{\partial \tau_{k}^{i}\left(y_{n,(i-1)}^{\left(k\right)}\right)}{\partial y_{n,(i-1)}^{\left(k\right)}}$ is the k×k Jacobian matrix resulting from the *i*-th transport map in the *k*-th sub-model $\tau_{k}^{i}$ at the *n*-th sample. Therefore, the calculation of $|detJ_{\tau_{k}}|$ becomes (I+1) individual $|detJ_{\tau_{k}^{i}}|$. In practice, $J_{\tau_{k}^{i}}$ is often constructed as a lower-triangular matrix to further simplify the calculation. By parameterizing $\tau_{k}=\tau_{k}\left(\left(u_{1},\ldots ,u_{k}\right);\theta_{k}\right)$, the model training process is solving for

$$\hat{\theta_{k}}=\underset{\theta_{k}\in \Theta_{k}}{\arg\min}\frac{1}{N}\sum_{n=1}^{N}-\left(\sum_{i=0}^{I}\log\left|detJ_{\tau_{k}^{i}}\left(y_{n,(i-1)}^{\left(k\right)};\theta_{k}\right)\right|\right)-\log p_{k}\left(\tau_{k}\left(\left(u_{n1},\ldots ,u_{nk}\right);\theta_{k}\right)\right)$$

At each *k*, the explicit form of $\tau_{k}^{0},\tau_{k}^{1},\ldots ,\tau_{k}^{I}$ and $\theta_{k}$ depends on parameterization. The entire autoregressive flow architecture will start from k=1 and proceed to the next sub-model until the *d*-th one finishes training.

In the next paragraph, we will introduce a parameterization of the transport maps using averaging beta distributions and a choice of the hidden variable. It will also be demonstrated how the training is completed based on the design and how the hidden variable facilitates the training for each sub-model.

### 2.2. Parameterization of Transport Map

Focusing on the *k*-th sub-model, the purpose of $\tau_{k}$ is to map uniformly distributed samples $\left(u_{1},\ldots ,u_{k}\right)$ to samples $\left(x_{1}^{\left(k\right)},\ldots ,x_{k}^{\left(k\right)}\right)$. A similar design as shown in Liu (2024) [24] is adopted. Starting from the base transformation, $\tau_{k}^{0}=F^{-1}$ is parameterized as the inverse Gaussian CDF $\Phi^{-1}$, which builds a projection from hypercubical ${\left[0,1\right]}^{k}$ to full space $R^{k}$. The following transport maps $\tau_{k}^{i},i>0$ perform projection from $R^{k}$ to $R^{k}$. To introduce the dependence structure into the samples and construct an autoregressive relationship, this method uses the form $\tau_{k}^{i}\left(y_{i-1}^{k}\right)=T_{k}^{i}\left(L^{ki}y_{i-1}^{k}+b^{ki}\right)$ to induce the variable correlations. Here, $T_{k}^{i}$ is an element-wise transform such that

$$T_{k}^{i}\left(z\right)=\left(T_{k1}^{i}\left(z_{1}\right),\ldots ,T_{kk}^{i}\left(z_{k}\right)\right),i>0,$$

where $z=(z_{1},\ldots ,z_{k})$ is a notation for any k-dimension vector. $L^{ki}$ is a lower-triangle square matrix, and $b^{ki}$ is a shift applied to each dimension. Given the properties of $L^{ki}$ and $T_{k}^{i}$, the lower-triangle structure is inherited by a Jacobian of $\tau_{k}^{i}$. With this parameterization, it can be derived that the $\log detJ_{\tau_{k}^{i}}$ is a summation of the log derivatives on each element and contribution of the main diagonal of $L^{ki}$

$$\log detJ_{\tau_{k}^{i}}\left(y_{i-1}^{\left(k\right)}\right)=\sum_{j=1}^{k}\log{\tilde{T}}_{kj}^{i}\left({\left(L^{ki}y_{n,(i-1)}^{\left(k\right)}+b^{ki}\right)}_{j}\right)+\log L_{jj}^{ki},$$

where ${\tilde{T}}_{kj}^{i}$ is the derivative of $T_{kj}^{i}$, $y_{ij}^{\left(k\right)}$ is the *j*-th element of $y_{i}^{\left(k\right)}$, and ${\left\{L_{jj}^{ki}\right\}}_{j=1}^{k}$ are the elements on the diagonal of $L^{ki}$. To ensure the inversibility of $\tau_{k}^{i}$, the ${\left\{L_{jj}^{ki}\right\}}_{j=1}^{k}$ has to be positive.

The $T_{k}^{i}$ introduces nonlinearity to the transport map by taking a sandwich form on each element

$$T_{kj}^{i}\left(z_{j}\right)=F^{-1}\circ \psi_{kj}^{i}\circ F\left(z_{j}\right),j\leq k$$

$F^{-1}$ is the base transformation, and $\psi_{kj}^{i}$ is a mapping from [0,1] to [0,1]. More specifically, we parameterize $\psi_{kj}^{i}$ to be the weighted average of a number of Beta distribution CDFs, i.e.,

$$\psi \left(v\right)=\sum_{s=1}^{S}\omega_{s}F_{beta}\left(v,\alpha_{s},\beta_{s}\right)$$

$F_{beta}\left(v,\alpha_{s},\beta_{s}\right)$ is the CDF of Beta distribution. $\left\{\alpha_{s}\right\},\left\{\beta_{s}\right\}$ are the pre-defined parameters. $\left\{\omega_{s}\right\}$ is a vector of weights summed up to 1. $\left\{\alpha_{s}\right\},\left\{\beta_{s}\right\}$ can be shared across all $\psi_{kj}^{i}$, but $\left\{\omega_{s}\right\}$ are unique for each $\psi_{kj}^{i}$.

In the *k*-th sub-model, the parameters to be determined are the L,b,ω in each transport map. Since we have parameterized all the steps, the optimization problem can be solved using the BFGS algorithm. A full expression of the objective function $KL(q_{\tau_{k}}||p_{k})$ can be found in Appendix A. The derivative of objective function over parameters $\frac{\partial KL(q_{\tau_{k}}||p_{k})}{\partial \theta }$ are complex since each parameter involves multiple transport maps. In practice, those derivatives are calculated using numerical techniques. The hyperparameters *I* and *S* control the depth of the neural architecture and the flexibility of the transport maps, respectively. Larger values will increase the precision of the approximation for any continuous target distribution.

### 2.3. Hidden Variables

From the parameterization above, we can observe that as the sub-model index *k* goes from 0 to *d*, the dimension of *L* and *b* are increasing 1 at a time. Since an autoregressive feature is preserved along the flows, the training on the *k*-th sub-model will only depend on the first *k* dimensions. When training the $\left(u_{1},\ldots ,u_{k}\right)$ to approximate $p_{k}$, we can take advantage of the result that $\left(x_{1}^{\left(k-1\right)},\ldots ,x_{k-1}^{\left(k-1\right)}\right)$ is already an approximation of the marginal distribution $p_{k-1}$. At the beginning of the training, the model can borrow the already optimized parameters $\theta^{k-1}$ for the initialization value of $\theta^{k}$. At each transport map i>0, we can fill in the upper left part of $L^{ki}$ with $L^{(k-1)i}$ and the first elements of $b^{ki}$ with $b^{(k-1)i}$. The weights in the Beta averaging can be carried over directly. The next sub-model solution will start from here and optimize $\theta^{k}$, then pass it to the next sub-model as the starting value again. By including the $\theta^{k}$ as the hidden variable and passing through the flow, we can speed up the training process and stabilize the results.

## 3. Simulation

Building on the methodology introduced in the previous section, we conduct numerical experiments to evaluate the practical performance of the proposed model. Specifically, we consider the two-dimensional banana-shaped normal distribution, a canonical example of a complex distribution with strong nonlinear dependencies, to assess the model’s effectiveness in sampling and integral estimation. Training datasets are generated using RQMC methods and used to fit the model with I=2, S=10 and a maximum of 150 iterations on each dimension. For comparison purposes, we trained another neural autoregressive flow on the training set generated by the vanilla MC method with the same set of hyperparameters. Both training sets have 1024 samples. Since the banana function is a two-dimension distribution (d=2), there are two sub-models in each method. As we have pointed out in the previous section, the first sub-model will minimize the KL divergence between the trained distribution and the marginal banana distribution on the first dimension. The second sub-model will utilize the result in the first dimension and approximate the joint target distribution on both dimensions.

**Figure 2:**
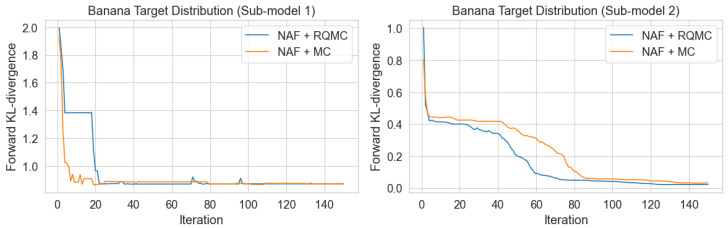
The trend in loss function as the number of iterations increases.

Figure 2 illustrates the progression of the forward KL divergence during training for both models as the number of iterations increases. In the first sub-model, where the marginal target distribution has a relatively simple structure, both methods exhibit rapid convergence. Although the NAF + MC method converges slightly faster than NAF + RQMC, both reach convergence within 20 iterations. This fast convergence can be attributed to the simplicity of the target distribution on the low dimension. In contrast, the second sub-model incorporates dependence structures into the training process, resulting in a slower convergence rate. Under this more complex joint distribution, the NAF + RQMC method converges more quickly than NAF + MC. While both approaches stabilize after approximately 100 iterations and ultimately attain similar final values, NAF + RQMC demonstrates greater efficiency in complex scenarios, achieving comparable performance with fewer computational resources.

**Figure 3:**
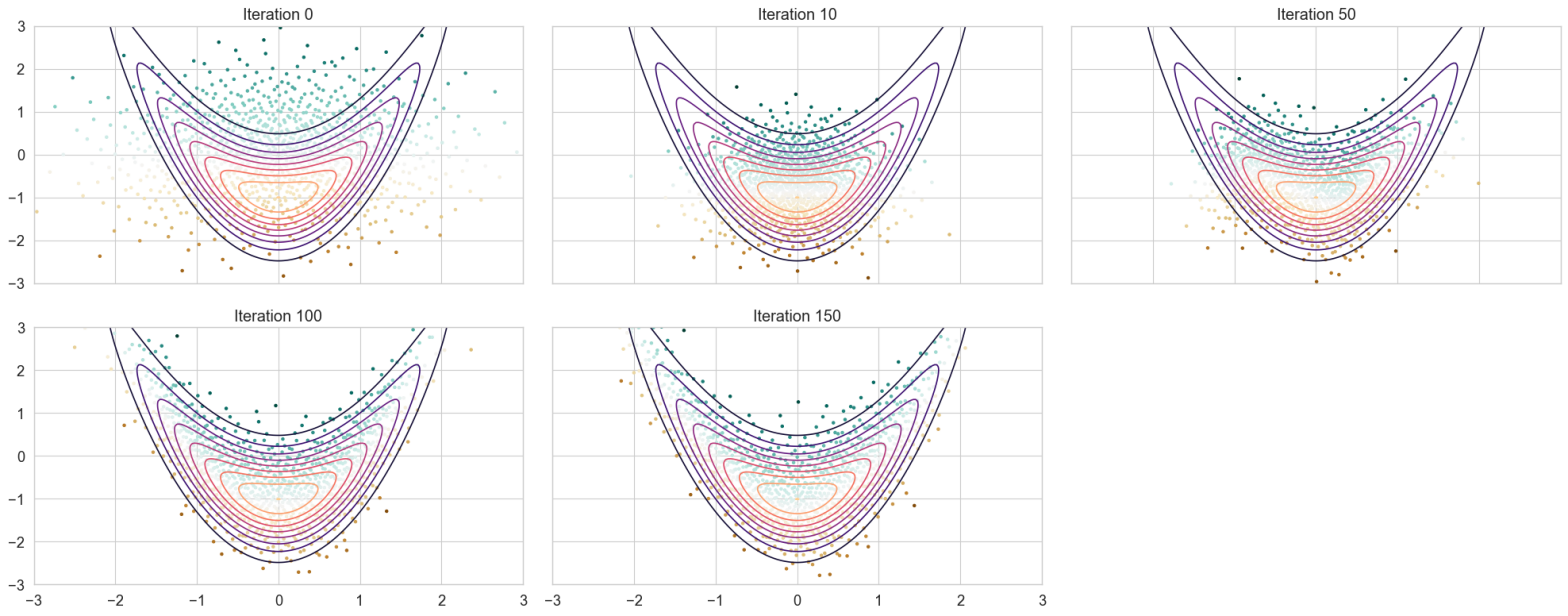
NAF+ RQMC fitting on 1024 samples.

**Figure 4:**
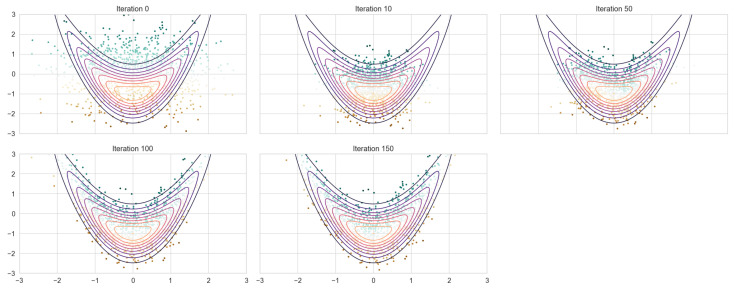
NAF+MC fitting on 1024 samples.

The trained model is then applied to an independent testing set generated via RQMC and MC according to their specific design. The resulting transformed samples approximate the banana-shaped distribution, as illustrated in Figure 3. As the number of iterations increases, the shape of the transformed samples gradually shifts from a circular form to the characteristic banana shape. In parallel, the empirical mean curve converges toward the true trajectory of the banana distribution. When compared to the neural autoregressive flow trained on Monte Carlo samples (NAF + MC) with the same hyperparameter, both approaches capture the target distribution well; however, the RQMC-based model (NAF + RQMC) exhibits superior performance in capturing the tail regions (Figure 4). The generated samples spread out more evenly around the shape of the target distribution instead of gathering in small clusters.

We further evaluate the model’s utility in integral estimation by comparing it with NAF + MC and a standard importance sampling method. Specifically, we estimate second-order moments: $E[x_{1}^{2}]$, $E[x_{2}^{2}]$, and $E[x_{1}^{2}+x_{2}^{2}]$. Each experiment is repeated 10 times, and the mean squared errors (MSEs) of the estimates are reported in Table 1. The proposed method achieves notable improvements in MSE over both baselines, demonstrating enhanced accuracy and stability in high-variance regions of the target distribution.

## 4. A-Share Data Application

This section presents a comprehensive empirical framework for modeling and simulating multivariate stock returns, grounded in the assumption of joint normality but emphasizing data-driven calibration and operational flexibility. Leveraging daily return data from Chinese A-shares (2015–2024), we construct annual target distributions using first-half-year data to generate synthetic samples for the second half. The model demonstrates robustness in capturing market dynamics, notably predicting the 2016 downturn with high accuracy. While the normal distribution provides tractability and theoretical justification, we critically examine its limitations regarding extreme events and propose pathways for incorporating alternative distributions. Operational details for sample generation and annual recalibration are thoroughly discussed. Empirical results validate the model’s efficacy in high-dimensional settings for applications in risk management and portfolio simulation.

The financial modeling of asset returns constitutes a cornerstone of modern portfolio theory (Markowitz, 1952) [25] and risk management (Jorion, 2006) [26]. A fundamental assumption pervasive in both theoretical and applied work posits that daily stock returns follow a normal distribution. This assumption, while frequently challenged by empirical observations of skewness and fat tails (Cont, 2001) [27], persists due to its mathematical tractability, the support offered by the Central Limit Theorem (CLT) for aggregate effects, and its foundational role in seminal frameworks like the Capital Asset Pricing Model (CAPM) (Sharpe, 1964) [28] and option pricing theory (Black & Scholes, 1973) [29]. Building on the literature, we integrate machine learning methods into the proposed model to forecast stock returns. This paper develops and empirically tests a pragmatic modeling framework centered on the multivariate normal distribution for portfolio returns, explicitly deriving its parameters—the mean vector and covariance matrix—from observed market data rather than imposing them theoretically. The primary objective is to generate synthetic return vectors that statistically mirror the properties of a specified target period, enabling robust applications in scenario analysis, portfolio stress testing, and forecasting.

### 4.1. Model Description

The model’s distinctiveness lies in its rigorous operationalization: annual recalibration using a fixed training window (January–June), explicit handling of a variable number of stocks, empirical validation against out-of-sample test data (July-December), and inherent flexibility to extend beyond the normal distribution assumption. We apply this framework to the dynamic and significant Chinese A-share market over a decade (2015–2024), encompassing diverse market regimes, to rigorously assess its performance and limitations. The model rests on the assumption that the vector of daily returns for a portfolio comprising N individual stocks follows an N-dimensional joint normal (multivariate normal) distribution. Formally, the return vector $r={\left[r_{1},r_{2},\ldots ,r_{N}\right]}^{T}$ is modeled as

$$r∼N_{N}\left(\mu ,\Sigma \right)$$

where $\mu ={\left[\mu_{1},\mu_{2},\ldots ,\mu_{N}\right]}^{T}$ is the vector of expected daily returns (mean vector). Σ is the N×N covariance matrix, capturing both individual stock volatilities ($\sigma_{i}^{2}=\Sigma_{ii}$) and the pairwise covariances ($\sigma_{ij}=\Sigma_{ij},i\neq j$) between stocks.

This assumption offers significant advantages: 1. Theoretical Underpinning: The CLT suggests that the net effect of numerous independent, random factors influencing stock prices may result in returns approximating normality, especially over shorter horizons like daily trading (Fama, 1965) [30]. 2. Mathematical Tractability: The multivariate normal distribution is exceptionally well-studied. Its properties are fully characterized by the first two moments (μ and Σ), and its probability density function (PDF) has a closed-form expression. Linear combinations of normally distributed returns remain normal, simplifying portfolio analysis. 3. Diversification Modeling: The covariance matrix Σ explicitly quantifies the co-movement between assets, which is fundamental to understanding and exploiting diversification benefits within a portfolio (Elton & Gruber, 1997 [31]). Portfolio variance depends directly on Σ.

We acknowledge, however, the well-documented empirical deviations from normality in financial returns, including skewness (asymmetric return distributions) and leptokurtosis (fatter tails than the normal distribution, indicating higher frequency of extreme events) (Mandelbrot, 1963 [32]; Fama, 1965 [30]). These deviations, particularly relevant during market crises, represent a key limitation addressed in Section 2.3 and the discussion of the results.

### 4.2. Empirical Derivation of the Target Function

Critically, the target multivariate normal distribution $N_{N}\left(\mu ,\Sigma \right)$ is not an arbitrary theoretical construct. It is derived empirically from observed market data. Specifically, for each calendar year and a selected portfolio of N stocks, the model utilizes daily return data exclusively from the first half of the year (January 1st to June 30th) as the training period.

From this training dataset, the sample estimates of the mean vector and covariance matrix are calculated: 1. Sample Mean Vector ($\hat{\mu }$): ${\hat{\mu }}_{i}=\frac{1}{T}\sum_{t=1}^{T}r_{i,t}$, where *T* is the number of trading days in the training period (approx. 125 days), and $r_{i,t}$ is the return of stock *i* on day *t*. 2. Sample Covariance Matrix ($\hat{\Sigma }$): ${\hat{\Sigma }}_{ij}=\frac{1}{T-1}\sum_{t=1}^{T}\left(r_{i,t}-{\hat{\mu }}_{i}\right)\left(r_{j,t}-{\hat{\mu }}_{j}\right)$.

These estimates define the target distribution for the year: $r∼N_{N}\left(\hat{\mu },\hat{\Sigma }\right)$. The probability density function (PDF) of this target distribution is

$$f\left(r\right)=\frac{1}{{\left(2\pi \right)}^{N/2}{\left|\hat{\Sigma }\right|}^{1/2}}\exp\left(-\frac{1}{2}{\left(r-\hat{\mu }\right)}^{T}{\hat{\Sigma }}^{-1}\left(r-\hat{\mu }\right)\right)$$

where $|\hat{\Sigma }|$ denotes the determinant of the estimated covariance matrix. This data-driven approach ensures the model is intrinsically calibrated to the specific statistical properties—volatilities, correlations, and average returns—observed in the market during the training window, maximizing its relevance for simulating conditions relevant to that period. The primary objective of the model training phase is to learn the target distribution $N_{N}\left(\hat{\mu },\hat{\Sigma }\right)$ sufficiently well to generate realistic synthetic return vectors (R). These synthetic samples mimic all the possible daily returns that could occur in the second half of the year.

### 4.3. Operational Process for Model Implementation

The model’s effectiveness hinges on its data-centric nature. Each calendar year constitutes a distinct modeling cycle: 1. Data Acquisition: Collect daily closing price data for the pre-selected N stocks for the period January 1st to June 30th. Calculate daily logarithmic returns: $r_{t}=\ln\left(P_{t}/P_{t-1}\right)$. 2. Parameter Estimation: Compute the sample mean vector $\hat{\mu }$ and sample covariance matrix $\hat{\Sigma }$ from the return data. 3. Target Function Construction:nDefine the target PDF f(r) using the estimated $\hat{\mu }$ and $\hat{\Sigma }$. 4. Annual Recalibration: This process repeats every year. Using new training data from January to June, new estimates ${\hat{\mu }}_{\mathrm{year}}$ and ${\hat{\Sigma }}_{\mathrm{year}}$ are computed, defining a new target distribution specific to that year’s observed first-half dynamics.

Recalibration is crucial for adapting to the non-stationary nature of financial markets. Volatility regimes shift (e.g., low-volatility vs. high-volatility periods), correlations between assets change due to evolving economic fundamentals or sector dynamics, and average returns fluctuate. Annual updates ensure the model reflects the prevailing market conditions relevant for simulating the upcoming second half of the year.

Once the target distribution is defined for a given year, the model parameters are trained to provide an approximation of the target distribution. Synthetic return vectors are generated using the trained parameters and a set of new RQMC samples. Key statistics (e.g., portfolio volatilities, individual stock return) derived from the synthetic data are compared to those from the training data.

### 4.4. Data Selection and Temporal Structure

To rigorously evaluate the model, we utilize daily stock return data from the Chinese A-share market, spanning a significant and turbulent decade from January 2015 to December 2024. This period offers a rich tapestry of market conditions: 2015–2016: massive speculative bubble and subsequent sharp crash (−40% in major indices), circuit breaker implementation and withdrawal, significant government intervention; 2017: periods of relative stability and growth, followed by escalating trade tensions impacting sentiment; 2018: the first wave of impact caused by trade tensions; 2019: recovery stage from the tension; 2020–2022: extreme volatility driven by the COVID-19 pandemic, regulatory crackdowns (e.g., tech, education sectors), and property market distress; and 2023–2024: recovery attempts amidst global economic uncertainty and domestic policy shifts.

This diversity provides a stringent test bed for the model’s ability to adapt to different regimes. The temporal structure is strictly maintained as follows. 1. Annual Splitting: Each year is divided into two distinct, non-overlapping periods. Training Period (Calibration): 1 January, y–30 June, y is used to estimate ${\hat{\mu }}_{y}$ and ${\hat{\Sigma }}_{y}$. Testing Period (Out-of-Sample Validation): July 1st, y–December 31st, y is used to assess the model’s predictive performance or the realism of its simulations compared to actual observed returns. 2. Fixed Cut-off: June 30th serves as a consistent, predetermined point for splitting the data, mimicking a realistic forecasting scenario where only historical data is available to inform predictions about the future (second half of the year).

### 4.5. Dynamic Stock Selection

Reflecting the practical realities of portfolio management, the number of stocks (N) and the specific constituents included in the portfolio analyzed are allowed to vary annually. Selection criteria can be based on the following: market capitalization, focusing on large-caps, mid-caps, or a mix; liquidity, ensuring sufficient trading volume to minimize microstructure noise; sector representation, aiming for diversification across economic sectors (financial, industrial, consumer staples, tech, etc.); index membership, selecting constituents of major indices like the CSI 300 or SSE 50; and data availability and quality, excluding stocks with excessive missing data or delistings during the period.

This dynamic selection strategy tests the model’s scalability and robustness. The dimensionality N can range from relatively small portfolios (e.g., N = 50) to large ones (e.g., N = 100 or more), challenging the parameter estimation process (especially covariance matrix estimation) and computational efficiency. It ensures the model is evaluated under conditions reflecting actual investment practice where portfolios are rebalanced and reconstituted periodically.

### 4.6. Empirical Results and Model Performance

**Figure 5:**
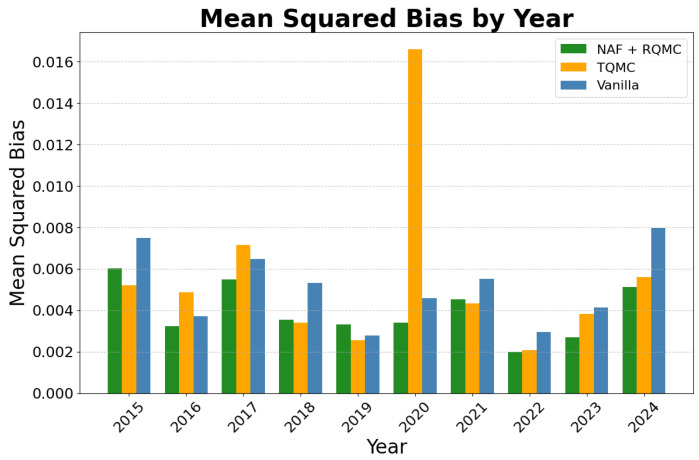
2015–2024 predicted A-share daily return mean squared bias.

**Figure 6:**
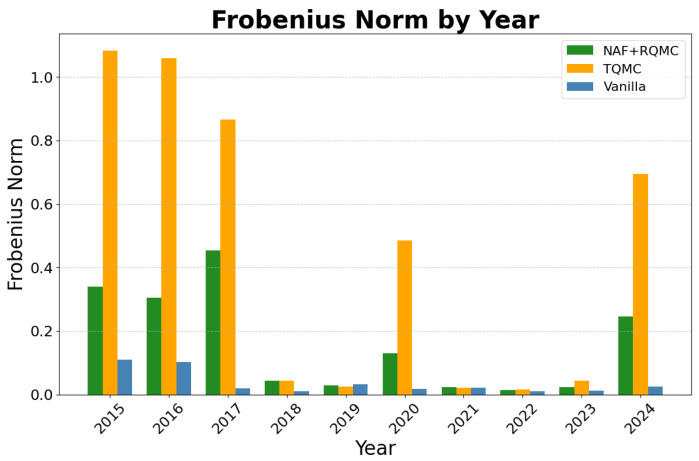
2015–2024 predicted A-share daily return variance Frobenius norm.

**Figure 7:**
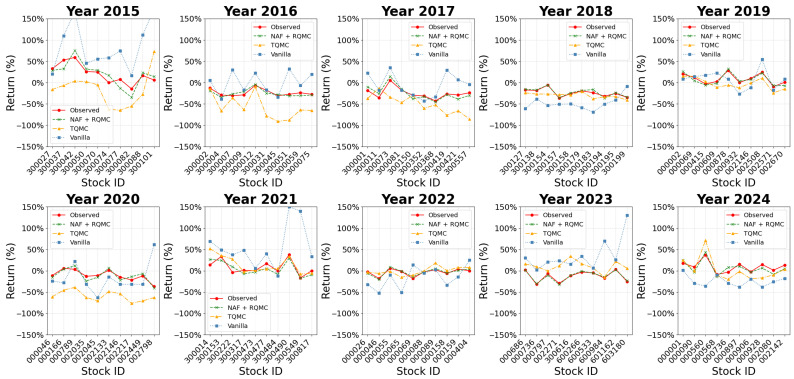
2015–2024 A-share half-year return prediction.

The model demonstrated strong operational performance and predictive capability across the entire 2015–2024 period. The NAF+RQMC model result was compared to the TQMC [24] and vanilla predictions. Vanilla prediction uses traditional methods for sample generation. Since we assumed normality in the daily stock return, the vanilla samples would be uniform samples drawn randomly and passed to the inverse of the distribution function to generate multivariate normal samples. Figure 5 presents the mean squared error of the predicted mean returns. The trained model predicts the daily returns with low bias consistently, and beats the other two methods in most years. This illustrates that the NAF + RQMC achieves higher accuracy than the other two methods. In some years, e.g., 2015 and 2024, the average bias is higher than the other years due to the fluctuation in the stock market. For most of the years, however, the prediction error is controlled within a low range. As for the variance in the structure of the selected stocks, Figure 6 shows the Frobenius norm of the variance matrix in the difference of the predicted data and the observed data. Vanilla prediction gives the lowest bias in variance term due to its simplicity in estimation. However, NAF + RQMC still controls this bias within a low range and outperforms the other method. Looking at the NAF+RQMC result during the known crackdown period, the variance matrix predicted is more biased than the other years, but overall, the error is under control. The sharp increases mostly result from the sharp change in the volatility of the stocks. Despite this, the predicted cumulative return is close to the observed ones in all years. This result is illustrated in Figure 7, which shows the predicted and observed cumulative returns in the second half of the year for some of the selected stocks. The model shows a satisfying performance in this mid-term prediction task and outperforms the TQMC and vanilla predictions. For each stock, the NAF + RQMC-predicted cumulative return is the closest to the actual one. Especially in the years 2016 and 2018, predicted returns and observed returns exhibit close agreement on very negative values. This reinforces the sharp changes in the years accordingly. Still, beyond 2015, the model demonstrated reliable performance throughout the decade. While the magnitude of predictive accuracy varied with market conditions (e.g., higher accuracy in trending markets, lower in highly volatile or sideways markets), the synthetic data consistently captured the prevailing correlation structures and volatility levels observed in the test periods.

### 4.7. Flexibility Beyond Normality

While the core model leverages the multivariate normal distribution, its operational structure is intentionally flexible to accommodate alternative continuous distributions better suited to capturing specific empirical characteristics. Fat Tails (Leptokurtosis): The multivariate Student’s t-distribution provides heavier tails than the normal while maintaining elliptical symmetry, often offering a better fit for financial returns (Praetz, 1972 [33]; Lange et al., 1989 [34]). Sample generation requires scaling normal vectors by the inverse square root of an independent chi-square variate. Asymmetry (Skewness): Skewed distributions like the multivariate skew-normal (Azzalini & Capitanio, 1999 [35]) or skew-t (Azzalini & Capitanio, 2003 [36]) can model asymmetric return patterns. Copula-based approaches (Nelsen, 2006 [37]; McNeil et al., 2015 [38]) offer immense flexibility by separating the modeling of marginal distributions (which can be non-normal) from the dependence structure (captured by the copula). Specific Applications: Distributions like the Exponential or Gamma might be relevant for modeling strictly positive quantities (e.g., trading volumes, time durations), while the F-distribution has applications in variance ratio tests. Models like GARCH (Bollerslev, 1986 [39]) or stochastic volatility directly address time-varying volatility clustering.

The choice of the normal distribution in this specific implementation is motivated by its simplicity, computational efficiency, theoretical grounding via the CLT for large diversified portfolios, and dominance in foundational financial theory. However, the framework readily allows for substituting the normal PDF and its corresponding sampling algorithm with those of an alternative distribution, enhancing the model’s ability to capture extreme events, asymmetry, or other non-normal features observed empirically, particularly in volatile markets or for specific asset classes. This represents a clear avenue for model enhancement.

## 5. Conclusions and Discussion

This paper proposed a transport quasi-Monte Carlo method built on a neural autoregressive flow architecture. The key idea of the model is to approximate the marginal density of the target distribution in the first few dimensions. As the model progresses, one more dimension is added at each step. The network construction of the transport map using normalizing flows within each sub-model and the flexible beta averaging bijection grants the model the ability to approximate any continuous target distribution. The unique feature of the lower triangular matrix introduces the dependence relationship among the elements and significantly reduces the computation burden for the optimization process. The utilization of parameters as hidden variables passing along the model further increases the efficacy and accuracy of the model. The choice of RQMC to generate initial samples from the hypercube ensures the sample coverage on the probability space, reducing the sample size required in each training and providing adequate samples at the tails of the target distribution. This is especially helpful when estimating integrals of high degrees or complex continuous distributions. In the simulation part, we compared the neural autoregressive flow applied to RQMC with the one applied to traditional Monte Carlo.

From the fitting results, it can be seen that the neural autoregressive flow pushes the samples to the designated shapes, while NAF + RQMC-generated samples show better performance at the edge of the target distribution compared to the NAF + MC. In the high-dimension optimization process, NAF + RQMC exhibits faster convergence. In terms of integral estimation, we calculated the first and second moments for both methods and added the estimation given by the trivial importance sampling method. We can observe improvements in the estimation of all moments in all dimensions in the proposed method. Especially compared with vanilla importance sampling, the MSE drops sharply by using the neural autoregressive flow architecture. In the A-share stock return prediction experiment, this model predicted the daily return and cumulative semiannual return for the stocks with high accuracy. The individual stock prediction also demonstrate the overall trend in the whole market. More importantly, the model captures the variance structures well even when the market fluctuates. All prediction errors are controlled within a low range. The model has good performance in mid-run predictions for the market.

There are still future works and potential improvements on this model. First, when dealing with a high-dimensional dataset, the model training becomes unstable as the sub-model goes to a high dimension. The optimization process may fail due to excessive training at the first few dimensions. A possible solution is to use an adaptive iterative step. By controlling the iteration numbers and changing the learning rate, we can let the training become more balanced on each dimension. Second, quasi-Monte Carlo usually has good convergence rates. By introducing a neural autoregressive flow on this task, it would be interesting to explore the rate of parameter convergence as the sample size grows. Last but not least, neural autoregressive flow builds a powerful architecture on the model; we would be excited to see other constructions of transport mapping on each of the sub-models.

## Figures and Tables

**Table 1:** Mean squared error (MSE) comparison for different integrals and methods.

Integral	NAF + RQMC	NAF + MC	IS + RQMC
$E[x_{1}]$	0.0008	0.0225	0.1347
$E[x_{2}]$	0.0127	0.1575	0.4336
$E[x_{1}+x_{2}]$	0.0178	0.0697	1.0069
$E[x_{1}^{2}]$	0.0913	0.1308	0.7775
$E[x_{2}^{2}]$	0.5729	0.8713	2.1667
$E[x_{1}^{2}+x_{2}^{2}]$	0.6600	0.9836	2.9241

## Data Availability

Data are contained within the article.

## Appendix A

Here, we show the full expression of loss function $\mathrm{KL}(q_{\tau_{k}}||p_{k})$ at the *k*-th sub-model:

$$\begin{array}{cc} \mathrm{KL}(q_{\tau_{k}}||p_{k})\; & =\frac{1}{N}\sum_{n=1}^{N}\log\frac{q_{\tau_{k}}\left(x_{n1}^{\left(k\right)},\ldots ,x_{nk}^{\left(k\right)}\right)}{p_{k}\left(x_{n1}^{\left(k\right)},\ldots ,x_{nk}^{\left(k\right)}\right)}\\ \; & =\frac{1}{N}\sum_{n=1}^{N}\left(\sum_{i=0}^{I}-\log\left|detJ_{\tau_{k}^{i}}\left(y_{n,(i-1)}^{\left(k\right)}\right)\right|\right)-\log p_{k}\left(x_{n1}^{\left(k\right)},\ldots ,x_{nk}^{\left(k\right)}\right) \end{array}$$

The transport maps involved are

$$\begin{array}{cc} \tau_{k}^{0}\left(u_{1},\ldots ,u_{k}\right)\; & =(F^{-1}\left(u_{1}\right),\ldots ,F^{-1}\left(u_{k}\right))\\ \tau_{k}^{i}\left(y_{n,(i-1)}^{\left(k\right)}\right)\; & =(T_{k1}^{i}{\left(L^{ki}y_{n,(i-1)}^{\left(k\right)}+b^{ki}\right)}_{1},\ldots ,T_{kk}^{i}{\left(L^{ki}y_{n,(i-1)}^{\left(k\right)}+b^{ki}\right)}_{k})\\ \; & =(F^{-1}\circ \psi_{k1}^{1}\circ F\left({\left(L^{ki}y_{n,(i-1)}^{\left(k\right)}+b^{ki}\right)}_{1}\right),\ldots ,\\ & \;\;\;F^{-1}\circ \psi_{kk}^{1}\circ F\left({\left(L^{ki}y_{n,(i-1)}^{\left(k\right)}+b^{ki}\right)}_{k}\right)),i>0 \end{array}$$

Since $L^{ki}$ are lower-triangle matrices and $T_{kj}^{i}$ are element-wise transforms, the Jacobian $J_{\tau_{k}^{i}}\left(y_{n,(i-1)}^{\left(k\right)}\right)$ are lower-triangles as well

$$\begin{array}{cc} detJ_{\tau_{k}^{i}}\left(y_{n,(i-1)}^{\left(k\right)}\right)\; & =\prod_{j=1}^{k}\frac{\partial {\left(\tau_{k}^{i}\left(L^{ki}y_{n,(i-1)}^{\left(k\right)}+b^{ki}\right)\right)}_{j}}{\partial y_{n,(i-1)j}^{\left(k\right)}}\\ \; & =\prod_{j=1}^{k}{\tilde{T}}_{kj}^{i}\left({\left(L^{ki}y_{n,(i-1)}^{\left(k\right)}+b^{ki}\right)}_{j}\right)\times L_{jj}^{ki}, \end{array}$$

where

$$\begin{array}{cc} {\tilde{T}}_{kj}^{i}\left({\left(L^{ki}y_{n,(i-1)}^{\left(k\right)}+b^{ki}\right)}_{j}\right)\; & =\frac{\partial F^{-1}\circ \psi_{kj}^{i}\circ F\left({\left(L^{ki}y_{n,(i-1)}^{\left(k\right)}+b^{ki}\right)}_{j}\right)}{\partial \psi_{kj}^{i}\circ F\left({\left(L^{ki}y_{n,(i-1)}^{\left(k\right)}+b^{ki}\right)}_{j}\right)}\times \\ & \;\;\;\;\;\frac{\partial \psi_{kj}^{i}\circ F\left({\left(L^{ki}y_{n,(i-1)}^{\left(k\right)}+b^{ki}\right)}_{j}\right)}{\partial F({\left(L^{ki}y_{n,(i-1)}^{\left(k\right)}+b^{ki}\right)}_{j})}\times \frac{\partial F({\left(L^{ki}y_{n,(i-1)}^{\left(k\right)}+b^{ki}\right)}_{j})}{\partial L^{ki}{\left(y_{n,(i-1)}^{\left(k\right)}+b^{ki}\right)}_{j}}\\ \; & ={F^{-1}}^{\prime }(\psi_{kj}^{i}\circ F\left({\left(L^{ki}y_{n,(i-1)}^{\left(k\right)}+b^{ki}\right)}_{j}\right)\times \\ & \;\;\;\;\;{\psi_{kj}^{i}}^{\prime }\left(F\left({\left(L^{ki}y_{n,(i-1)}^{\left(k\right)}+b^{ki}\right)}_{j}\right)\right)\times F^{\prime }\left({\left(L^{ki}y_{n,(i-1)}^{\left(k\right)}+b^{ki}\right)}_{j}\right) \end{array}$$
